# Effects of winter indoor environment on the skin: Unveiling skin condition changes in Korea

**DOI:** 10.1111/srt.13397

**Published:** 2023-06-13

**Authors:** Eun Hye Park, Da Jung Jo, Hyo Won Jeon, Seong Jin Na

**Affiliations:** ^1^ Research institute Celltem Pharm Co., Ltd. Seoul South Korea

**Keywords:** ceramide NP, indoor environment, normal skin, skin barrier, skin characteristics, winter season

## Abstract

**Background:**

In Korea, winter can cause skin dryness due to low relative humidity (RH); moreover, indoor heating devices promote moisture loss and air pollution. If dryness persists, dead skin cells accumulate, leading to skin problems; therefore, careful skin care is required. This study aimed to compare changes in skin conditions when exposed to an indoor environment for a short period of 6 h in winter, and to suggest proper winter skin care practices.

**Methods:**

A randomized, split‐face clinical study was conducted in which healthy female participants with normal skin were exposed to an indoor environment with a heater turned on for a short period at least 6 h per day in the winter season, and cream was applied to one side of the face. Skin temperature, hydration, sebum, transepidermal water loss (TEWL), elasticity, texture, pores, redness, and wrinkles were measured at the treated and nontreated sites.

**Results:**

After 6 h of exposure, skin temperature, pores, roughness, redness, and wrinkles significantly increased (*p* < 0.05) on the face, whereas TEWL significantly increased on the forearm (*p* < 0.05). However, sebum secretion appeared to function as a barrier to maintain homeostasis in the facial skin. Elasticity, pores, texture, and wrinkles in the cream‐treated ceramide site improved compared to those in the nontreated site (*p* < 0.05). The moisture content was also significantly higher in the forearm (*p* < 0.05).

**Conclusion:**

Changes in skin parameters of participants with healthy skin were observed even after short‐term exposure to an indoor environment in winter. Creams containing ceramide maintain skin homeostasis and protect the skin barrier; therefore, it is recommended to use such creams to prevent skin damage and maintain healthy skin, particularly during prolonged exposure to indoor environments during winter.

## 1 INTRODUCTION

As per the Korean Statistical Information Service, the country has the longest working hours among OECD countries, with a total of 1928 h as of 2021. Many office workers in Korea complain about feeling dry air during winter. Hot air blown out by heating devices used in offices can strip the skin of moisture; moreover, providing proper ventilation may be difficult in cold weather. This can result in exposure to indoor dust and dry environments. If this environmental exposure persists, it can lead to skin stress and may accelerate the aging process.^1^ Several studies have reported that prolonged exposure to extremely low humidity can lead to skin conditions, such as contact dermatitis in workers.^2^, ^3^ A previous study demonstrated that abrupt fluctuations in humidity can have an adverse effect on skin barrier function, potentially leading to skin diseases.^4^ According to Jang et al.,^5^ women over 30 years of age experienced a greater reduction in skin elasticity as a result of repeated alterations in temperature and humidity.

Changes in temperature also affect the skin. Many studies have reported the relationship between temperature changes and skin hydration as well as TEWL. Temperature and TEWL showed a positive correlation.^6^, ^7^ However, the relationship between relative humidity and TEWL has not been clarified in previous papers.^8^, ^9^ Numerous studies have examined seasonal changes in skin characteristics. A previous study found that the incidence of atopic skin exacerbation, a condition characterized by dry, itchy, and inflamed skin, is highest in winter and spring. Specifically, the incidence rates of atopic skin exacerbation were 25%, 19%, 11%, and 36% in the spring, summer, autumn, and winter seasons, respectively.^10^ According to Qiu et al.,^11^ there was no notable difference in aging parameters between summer and winter in relation to age. However, changes were observed in certain functional criteria, such as skin color, pigmentation, sebum secretion, and hydration. In another study, men showed significant changes in wrinkle depth during winter season.^12^

People commonly experience skin problems and discomfort during winter, especially when exposed to dry indoor environments. However, only few studies in Korea have investigated the effects of winter conditions in an office environment on normal skin without skin diseases. Therefore, in this study, changes in skin characteristics were investigated by controlling the exposure environment for 6 h. In addition, improvements in the area where a ceramide‐containing cream was applied were investigated.

## 2 METHODS

### 2.1 Participants and environmental condition

We recruited and selected Korean women between the ages of 20 and 59 years with normal skin type. A normal skin type was judged based on a sebumeter's measured value of 30−60.^13^ This study was conducted in accordance with the ethical standards of the Declaration of Helsinki and approved by the appropriate Institutional Review Board (P01‐202301‐01‐037). The purpose and method of the study and possible adverse reactions were explained to all participants who voluntarily partook in this study and filled out an informed consent form. The number and age of the participants in each region are summarized in Table 1.

**TABLE 1:** Number of participants (*N*), age, and sebum content at baseline point.

Measurement site	*N*	Age (mean ± SD)	Sebum (mean ± SD)
Face	20	41.90 ± 9.62	43.18 ± 12.84
Forearm	12	34.83 ± 8.85	–

**FIGURE 1:**
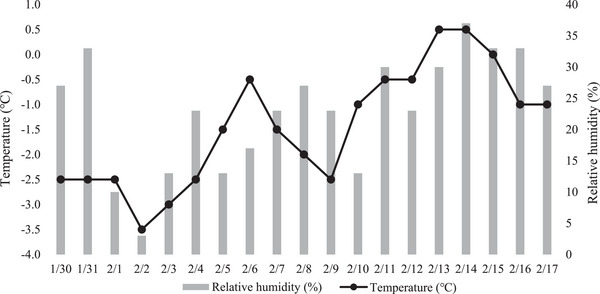
Average daily temperature (T) and relative humidity (RH) during the study period.

To achieve exposure to a dry indoor environment in winter, the study was conducted in January and February, when humidity was low, according to the Korea Meteorological Administration website (https://www.weather.go.kr/). During the study period, the minimum and maximum mean temperatures in Seoul were −5.68°C and 2.79°C, respectively, and humidity was 23.05% (Figure 1). The participants washed their faces and forearms with the cleansing foam in a laboratory equipped with constant temperature and humidity conditions (room temperature 20°C–24°C, humidity 45∼55% RH) and waited for 30 min for skin stabilization.

We evaluated the skin temperature, hydration, sebum, TEWL, elasticity, pores, texture, wrinkles, and redness on the participants’ faces and forearms before exposure (baseline) and 1 and 6 h after exposure. The participants waited for 6 h in an area maintained at a temperature of 25 ± 1°C and a humidity of less than 20% that was achieved by using a heater until the study was concluded.

### 2.2 Treatment

Facial creams were selected to investigate their effectiveness in managing potential skin changes caused by indoor conditions during winter. The key ingredients of the creams include ceramide NP, ectoin, glycerin, trehalose, and 2,3‐butanediol to improve the skin barrier and moisturize it.

After the baseline measurement, approximately 0.3 g of cream was applied to one side of the face (randomized among the participants). For the forearm, 12 μL of cream was applied to the treated site in a 2 × 2 cm area (randomization) and the sleeve was rolled up to allow the area to be exposed to the environment.

### 2.3 Measurement of skin biophysical parameters

Skin parameters, such as temperature, hydration, sebum, TEWL, elasticity, pores, texture, wrinkles, and redness were measured using various devices in a noninvasive method.

The skin temperature in the forehead area was recorded using a noncontact IR thermometer (HuBDIC Co., Ltd., South Korea). Skin hydration, sebum, TEWL, and skin elasticity were measured on the cheek using Corneometer^®^ CM 825, Sebumeter^®^ SM815, Tewameter TM HEX, and Cutometer MPA580 devices (C+K, Köln, Germany), respectively. Skin hydration and TEWL were additionally measured on the forearm because the external influence was minimized and areas with less sweat or hair could produce accurate and reliable results. Cheek skin redness, pores, texture, and wrinkles were measured using an Antera 3D CS (Miravex, Ireland).

### 2.4 Statistical analysis

All results are expressed as mean ± standard deviation (SD), and data were calculated using SPSS Statistics version 28.0 (IBM Corp., USA). Normality was assessed using the Shapiro–Wilk test for all data. Data for comparing the baseline and postexposure time points were analyzed using paired *t* tests (parametric) and Wilcoxon signed‐rank tests (nonparametric). Changes in skin characteristics between the treated and nontreated sites were compared using RMANOVA. The relationship between skin parameters was analyzed using Pearson's correlation coefficient test. Differences were considered statistically significant at *p* < 0.05.

## 3 RESULTS

**FIGURE 2:**
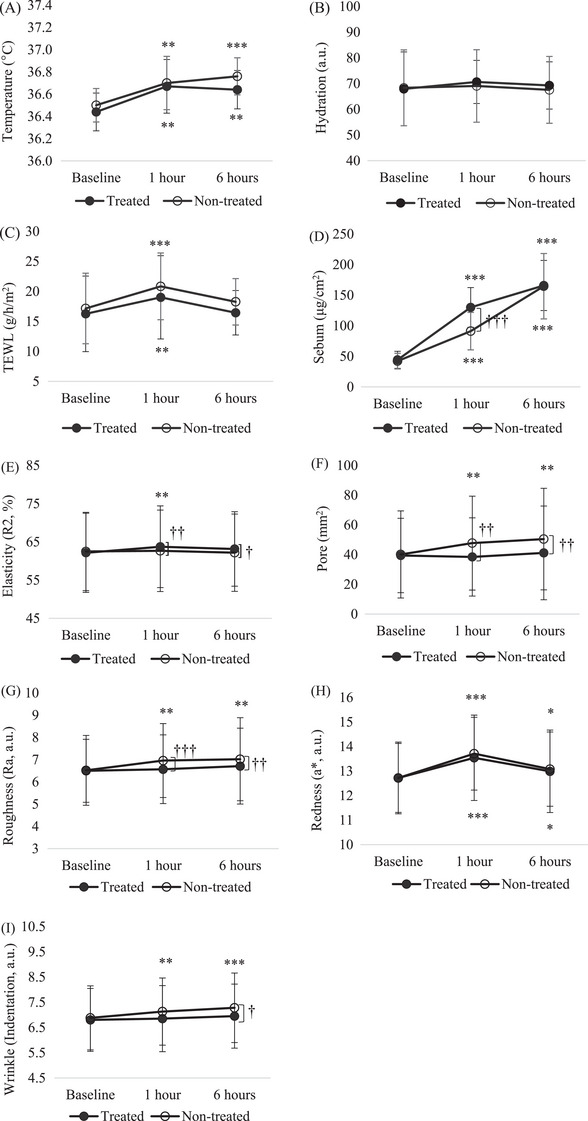
Comparison of skin parameters of the treated and nontreated site on the face before and after 1 and 6 h of exposure to an indoor environment in winter. (A) skin temperature, (B) hydration, (C) TEWL, (D) sebum, (E) elasticity, (F) pore, (G) roughness, (H) redness, and (I) wrinkles were evaluated. *p < 0.05, **p < 0.01, ***p < 0.001 baseline vs. 1 and 6 h postexposure time points. ^†^ *p* < 0.05, ^††^ *p* < 0.01, ^†††^ *p* < 0.001. nontreated vs. treated site.

According to the indoor environment exposure time in winter (temperature, 25 ± 1°C; humidity < 20%), the results of skin parameters (temperature, moisture, sebum, TEWL, elasticity, wrinkles, pores, texture, and redness) are shown in Figure 2.

The skin temperature of the nontreated site gradually increased by 0.54% and 0.73% after exposure for 1 h (*p* < 0.01) and 6 h (*p* < 0.001), respectively. The skin temperature of the treated site significantly increased by 0.62% within 1 h and was maintained at 0.55% for up to 6 h (*p* < 0.01, Figures 2A and 4A).

**FIGURE 3:**
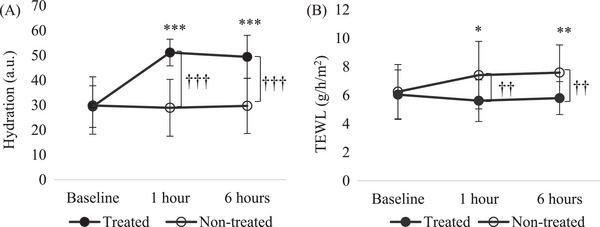
Comparison of skin parameters of the treated and nontreated site on the forearm before and after 1 and 6 h of exposure to the indoor environment in winter: (A) hydration and (B) TEWL. *p < 0.05, **p < 0.01, ***p < 0.001 baseline vs. 1 and 6 h postexposure time points. ^†^ *p* < 0.05, ^††^ *p* < 0.01, ^†††^ *p* < 0.001, nontreated vs. treated site.

**FIGURE 4:**
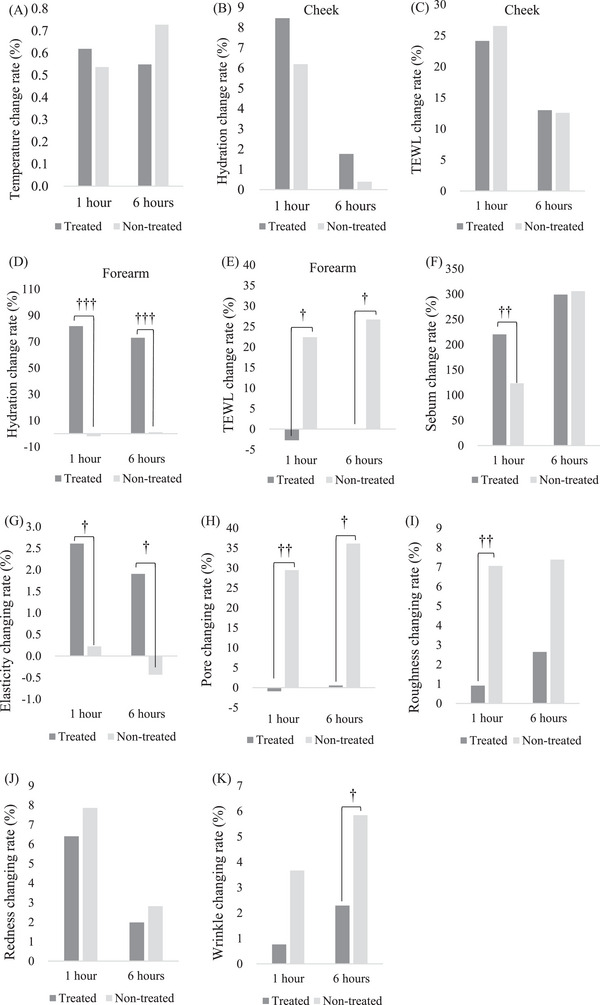
Rate of change of skin parameters after 1 and 6 h of exposure compared to baseline in treated and nontreated areas; (A) temperature, (B) hydration on the cheek, (C) TEWL on the cheek (D) hydration on the forearm, (e) TEWL on the forearm, (F) sebum, (G) elasticity, (H) pore, (I) roughness, (J) redness, (K) wrinkle were evaluated. ^†^ *p* < 0.05, ^††^ *p* < 0.01, ^†††^ *p* < 0.001. nontreated vs. treated site.

Regarding hydration and TEWL, compared to the baseline, there was no significant difference between the treated and nontreated sites after 6 h of exposure (Figures 2B and C and 4B and C). However, when hydration and TEWL were measured equally on the forearm, the results were completely different from those measured on the cheek. Hydration of the nontreated site on the forearm did not change significantly until after 6 h of exposure. The rate of change at the nontreated site was 1.11%. That of the treated site significantly increased by 81.81% and 73.02% after 1 and 6 h of exposure, respectively (*p* < 0.001). After exposure for 1 and 6 h, the increase in hydration at the treated site was significantly higher than that at nontreated sites (*p* < 0.001, Figures 3A and 4D). The TEWL of the nontreated site in the forearm significantly increased by 22.40% and 26.70% from 1 h (*p* < 0.05) to 6 h of exposure (*p* < 0.01), respectively. That of the treated site decreased slightly by 2.74% and 0.06% from 1 to 6 h of exposure, respectively. After exposure for 1 and 6 h, the decrease in TEWL at the treated site was significantly higher than that at the nontreated site (*p* < 0.01, Figures 3B and 4E).

Sebum at both sites significantly increased after 1 and 6 h of exposure (*p* < 0.001). After 1 h of exposure, sebum content at the treated site was significantly higher than that at the nontreated site (*p* < 0.001, Figures 2D and 4F).

The skin elasticity of the treated site significantly increased by 2.61% when exposed for 1 h (*p* < 0.01), whereas that of the nontreated site did not change significantly until after 6 h of exposure. After exposure for 1 and 6 h, the increase in elasticity at the treated site was significantly higher than that at the nontreated site (*p* < 0.05, Figures 2E and 4G).

The skin pore of the nontreated site significantly increased by 29.45% and 36.12% when exposed for 1 and 6 h, respectively (*p* < 0.01), and those of the treated site did not significantly change until after 6 h of exposure. After 1 and 6 h of exposure, the number of pores in the treated site was significantly lower than that in the nontreated site (*p* < 0.01, Figures 2F and 4H).

The skin roughness of the nontreated site significantly increased by 7.06% and 7.38% when exposed for 1 and 6 h, respectively (*p* < 0.01), and that of the treated site did not significantly change until after 6 h of exposure. After 1 h (*p* < 0.001) and 6 h (*p* < 0.01) of exposure, the increase in roughness at the treated site was significantly lower than that at the nontreated site (Figures 2G and 4I).

Skin redness at both sites increased significantly after 1 h (*p* < 0.001) and 6 h (*p* < 0.05) of exposure. However, there were no differences between the two sites until after 6 h of exposure (Figures 2H and 4J).

Skin wrinkles at nontreated sites significantly increased by 3.67% and 5.84% when exposed for 1 h (*p* < 0.01) and 6 h (*p* < 0.05), respectively; however, those at the treated site did not significantly change until after 6 h of exposure. Furthermore, after 6 h of exposure, the increase in wrinkles at the treated site was significantly lower than that at the nontreated site (*p* < 0.05, Figures 2I and 4K).

Table 2 shows the relationships between skin parameters that can affect the skin barrier after 6 h of exposure to indoor environments in winter between the treated and nontreated sites. A significant positive correlation between hydration and elasticity was found at the treated site where the ceramide cream was applied (*p* < 0.01). A significant positive correlation was found between TEWL, pore size, and roughness at the nontreated sites (*p* < 0.05). In contrast, the treated sites showed a positive correlation only between TEWL and pore (*p* < 0.05).

**TABLE 2:** Correlation of skin parameters after 6 h of exposure to an indoor environment in winter.

	Treated site				Nontreated site			
	Temperature	Hydration	TEWL	Sebum	Temperature	Hydration	TEWL	Sebum
Temperature	1.000	0.054	0.453*	−0.198	1.000	−0.238	0.354	−0.142
Hydration	0.054	1.000	0.060	−0.316	−0.238	1.000	0.123	0.110
TEWL	0.453*	0.060	1.000	−0.123	0.354	0.123	1.000	−0.175
Sebum	−0.198	−0.316	−0.123	1.000	−0.142	0.110	−0.175	1.000
Elasticity	0.000	0.687**	‐0.324	−0.113	−0.229	0.349	0.087	−0.228
Pore	0.375	−0.346	0.477*	0.202	0.365	0.074	0.508*	−0.187
Roughness	0.184	−0.362	0.239	0.369	0.388	0.078	0.513*	−0.224
Redness	0.174	0.124	−0.284	0.270	0.161	0.163	−0.284	0.275
Wrinkle	0.455*	0.062	0.316	0.153	−0.265	0.355	−0.088	0.409

* The correlation is significant at the 0.05 level (2‐tailed).

** Correlation is significant at the 0.01 level (2‐tailed).

## 4 DISCUSSION

In Korea, which has four distinct seasons, many people complain of dry skin due to office heaters every winter. Therefore, we aimed to investigate the effect of indoor environmental exposure on the skin using a noninvasive method and to determine whether these changes could be improved by applying a ceramide‐containing cream.

The skin temperature appeared to be affected by an increase in the ambient temperature owing to the use of heaters. The body maintains a constant skin temperature to regulate its internal environment, which is essential for homeostasis. Skin temperature also exhibits a high correlation with the temperature of the surrounding environment.^9^ In this study, although there was no difference between the two sites, the treated site maintained a relatively low skin temperature.

Skin redness also appears to be affected by changes in skin temperature. Several studies have shown that skin temperature and redness are positively correlated, as capillaries and blood flow change with ambient temperature.^14^

Hydration and TEWL in the face did not change significantly with exposure time. This suggests that the participant had a normal healthy skin barrier and adequate sebum secretion, which helped to maintain normal levels of skin hydration and TEWL on the face. However, hydration and TEWL in the forearm showed the opposite results. After 6 h of exposure, hydration decreased rapidly and TEWL significantly increased. This is because this area is less affected by the secretion of sebum and sweat.^15^ Furthermore, hydration and TEWL at the treated site in the forearm were significantly improved.

In many studies, the skin barrier has been improved by the application of products that contain ceramide to dry skin; however, there are few studies that show its effect on healthy normal skin.^16^

One hour after cream application, the sebum content of the skin increased significantly because of the lipid components present in the cream. However, there was no significant difference in sebum content between the cream‐treated and nontreated sites after 6 h. This is because even the nontreated site can protect the skin barrier during daytime by secreting sebum with a content similar to that of the treated site. According to Boer et al.,^17^ the most important biophysical parameters that determine the condition of the skin barrier are the skin pH, epidermal hydration, TEWL, and sebum secretion.

An increase in skin temperature can cause the pores to expand and expose the skin to dry air, especially in an enclosed environment, making the skin rough and prone to wrinkles. This can lead to skin fatigue and eventually decreased skin elasticity.^5^, ^18^

Hydration and elasticity were positively correlated at the treated site, while TEWL was positively correlated with pores and roughness at the nontreated site. The healthy skin of the participants maintained its barrier function through appropriate sebum secretion; however, this could still affect various aging‐related parameters. Creams containing ceramide were found to improve the elasticity, porosity, roughness, and wrinkling of the skin.

Therefore, in this study, continuous exposure to indoor environments in Korea during winter was observed to cause aging. It is suggested that this problem can be prevented by applying a ceramide cream, which improves the skin barrier.

## 5 CONCLUSIONS

In this study, we investigated the impact of normal facial skin when exposed to a high‐temperature, low‐humidity indoor environment for a short period during winter. Our findings revealed that after 6 h of exposure, skin temperature, roughness, redness, and wrinkles increased on the facial skin, whereas hydration and TEWL remained unchanged. In addition, the forearm area showed a significant decrease in hydration and an increase in TEWL. However, using creams containing 5% ceramide significantly increased hydration and decreased TEWL in the forearm area. Moreover, cream application improved the facial skin elasticity, pore size, roughness, and wrinkles. These results indicate that even individuals with normal skin experience adverse effects when exposed to indoor winter environments for a brief period. The use of a cream containing ceramide could be an effective solution to address these issues.

## CONFLICT OF INTEREST STATEMENT

The authors declare no conflict of interest.

## Data Availability

The data supporting the findings of this study are available from the corresponding author upon reasonable request.
